# Intermittent Fasting: Health Impacts and Therapeutic Potential

**DOI:** 10.3390/nu18101583

**Published:** 2026-05-15

**Authors:** Cristina Manuela Drăgoi, Alina Crenguța Nicolae

**Affiliations:** Department of Biochemistry, Faculty of Pharmacy, Carol Davila University of Medicine and Pharmacy, 6, Traian Vuia St., 020956 Bucharest, Romania

Intermittent fasting (IF) has emerged as one of the most intensively investigated dietary strategies of the past decade. Unlike classic continuous caloric restriction, IF imposes structured cycles of fasting and controlled eating, thereby engaging a distinct suite of adaptive cellular responses. These include the activation of autophagy, modulation of circadian metabolic rhythms, reduction in systemic inflammation and oxidative stress, and optimization of mitochondrial biogenesis [1,2,3,4]. The metabolic reprogramming induced by periodic nutrient deprivation has been associated with improvements in insulin sensitivity, body weight regulation, lipid profiles, and cardiometabolic risk markers across a range of preclinical and clinical settings [5,6,7,8].

Notwithstanding this progress, substantial knowledge gaps persist. The mechanistic pathways through which IF exerts organ-specific effects, particularly in the central nervous system, the gut–microbiome axis, and the tumor microenvironment, remain incompletely characterized. Similarly, questions around optimal fasting protocol selection, long-term adherence, and population-specific safety profiles (notably in cancer patients, older adults, and individuals with eating disorders) have yet to be answered through adequately powered, rigorously designed clinical trials [9,10,11,12,13].

To address these gaps, we launched this Special Issue of *Nutrients*, “Intermittent Fasting: Health Impacts and Therapeutic Potential”, and the response from the international scientific community was encouraging: seven contributions were ultimately accepted following rigorous peer review, spanning appetite mechanisms, endocrinology, gut microbiology, cognitive neuroscience, oncology, and integrative nutrition.

A pivotal research article by Breit et al. examined the secondary endpoints of a 12-month randomized behavioral weight loss trial comparing 4:3 IF (three non-consecutive fasting days per week providing ~25% of energy requirements) with daily caloric restriction (DCR) [14]. Analyzing appetite hormones and eating behavior questionnaires in 60 adults with overweight or obesity, the investigators found that 4:3 IF produced favorable modulation of ghrelin and peptide YY relative to DCR, attenuating the compensatory increases in hunger that typically accompany sustained energy restriction. Furthermore, participants in the IF arm reported less dietary preoccupation and improved cognitive restraint scores at 12 months. These findings suggest that the inherent diet flexibility of alternating feast and fast cycles may help circumvent the maladaptive behavioral adaptations that undermine long-term adherence to continuous restriction. This is a clinically important distinction given that weight regain remains the most common outcome of conventional dietary interventions.

Karras et al. contribute a pilot observational study examining leptin and interleukin-6 (IL-6) concentrations in vitamin D-deficient overweight Orthodox nuns from Northern Greece who adhere to the Athonian fasting calendar, a rigorous traditional regimen that prohibits meat, dairy, and often fish on approximately 180 days per year [15]. The results revealed that, despite concomitant vitamin D insufficiency, leptin levels were significantly associated with IL-6 in a pattern modulated by body mass index and fasting adherence. This study is notable for situating IF research within an ecological and cultural context that is frequently overlooked in laboratory-centric investigations: it demonstrates that real-world religiously motivated fasting practices can serve as natural experiments for elucidating the interplay of adipose tissue signaling and subclinical inflammation. The vitamin D deficiency documented in this cohort additionally points to the importance of micronutrient surveillance in individuals practicing restrictive dietary regimens.

An innovative research article by Naseri et al. explored a largely uncharted intersection between environmental exposure and nutritional intervention [16]. Using electroencephalography (EEG) to quantify cortical activity in healthy adults during and after a two-hour cooking session generating ultrafine aerosols, the authors observed measurable perturbations in alpha and beta band power consistent with transient neuroinflammatory responses. Critically, a post-exposure caloric restriction protocol attenuated these EEG alterations, implicating energy deprivation-induced anti-inflammatory and antioxidant pathways, including Nrf2 activation and BDNF upregulation, as potential mediators of neuroprotection against inhaled toxicants. While preliminary, this study broadens the conceptual scope of IF research by suggesting that fasting-related signaling may confer resilience against environmentally derived neurological events, a hypothesis that merits prospective validation.

The rapidly evolving field of fasting oncology was surveyed by Wen et al. in a comprehensive scoping review covering fasting-mimicking diets, short-term fasting before and after chemotherapy, and prolonged fasting protocols in cancer patients and survivors [17]. Synthesizing evidence from 38 clinical studies across multiple cancer types, the authors identify consistent signals for reduced chemotherapy-related toxicity, improved quality of life scores, and favorable modulation of insulin-like growth factor 1 (IGF-1) and glucose-to-ketone ratios in patients undergoing structured fasting. However, the review also underlines the substantial heterogeneity in fasting protocols, patient populations, and outcome measures across trials and the absence of phase III randomized controlled evidence supporting fasting as a standalone anti-tumor intervention. The authors advocate for standardized, multi-center trial designs and emphasize the necessity of oncology-specific safety monitoring frameworks before IF can be integrated into routine cancer care.

Draicchio and Axen published a thoughtful narrative review interrogating whether the cognitive benefits attributed to IF in experimental studies are mechanistically specific to fasting or simply secondary to energy restriction and adipose tissue loss [18]. Through a critical appraisal of human randomized trials and animal mechanistic data, the authors found that, while both IF and continuous caloric restriction improve cognitive outcomes in metabolically compromised individuals, IF-specific mechanisms (including episodic ketogenesis, time-restricted feeding-mediated circadian alignment, and intermittent BDNF surges) may confer cognitive benefits that are not fully recapitulated by isocaloric daily restriction. The review highlights the fact that most human studies are underrepresented and of short duration and calls for trials that deploy sensitive neuropsychological batteries and neuroimaging endpoints alongside standard metabolic measures.

Dumitrescu et al. offered an integrative review tracing the agronomic, culinary, biochemical, and neurological dimensions of olive oil (OO) within the broader context of the Mediterranean diet and IF [19]. Drawing on polyphenol biochemistry, particularly the anti-inflammatory and antioxidant properties of oleocanthal, oleuropein, and hydroxytyrosol, the authors construct a mechanistic argument for synergism between OO consumption and IF-induced metabolic adaptation. The reviewed topics range from soil composition and olive cultivar selection as determinants of polyphenol content through consumer behavior and label literacy to the neurocognitive correlates of OO-enriched dietary patterns. The breadth of this contribution exemplifies the interdisciplinary approach that characterizes contemporary nutritional science and reinforces the notion that the health effects of IF cannot be evaluated in isolation from the qualitative composition of eating windows.

Closing out this Special Issue, Zhang et al. synthesize evidence on the combined effects of IF and probiotic supplementation on gut microbial ecology and glycemic control in type 2 diabetes mellitus (T2DM) [20]. Their narrative review details how IF promotes microbial diversity and enriches short-chain fatty acid (SCFA)-producing taxa such as Akkermansia muciniphila, Faecalibacterium prausnitzii, and Bifidobacterium spp., while probiotics provide complementary colonization of beneficial strains that reinforce epithelial barrier function and attenuate lipopolysaccharide-driven systemic inflammation. The synergistic glycemic benefits reported in pilot trials, including reductions in fasting glucose, HbA1c, and HOMA-IR, position the IF–probiotic combination as a mechanistically coherent and clinically attractive adjunct to standard T2DM pharmacotherapy, though the authors appropriately caveat that strain-specific, protocol-matched randomized trials are needed to translate these observations into clinical guidelines.

Across the published contributions, several converging themes emerge that collectively delineate the current state of IF science and its trajectory. First, the metabolic and hormonal versatility of IF is evident: from ghrelin suppression and appetite normalization to IGF-1 modulation and microbiome reshaping, studies confirm that IF exerts pleiotropic effects that are not reducible to simple caloric deficit. The mechanistic diversity of IF responses, spanning circadian biology, cellular autophagy, SCFA metabolism, and neuroprotective signaling, suggests that different fasting protocols may be optimally matched to specific therapeutic goals and patient profiles [21,22,23,24,25,26].

Secondly, these contributions collectively highlight the importance of dietary context. The synergies identified between IF and high-quality dietary components, notably the polyphenol-rich olive oil of the Mediterranean diet and microbiome-modulating probiotics, argue against the isolated study of fasting timing in nutritional epidemiology. Future research must account for the qualitative composition of eating windows as a critical co-determinant of outcomes [27,28,29,30,31,32].

In addition, real-world and culturally situated fasting practices, as illustrated by the Athonian calendar study, offer a valuable and underexplored reservoir of observational data. These natural experiments can complement controlled trials by providing evidence on long-term adherence and population-level safety in fasting populations who have practiced structured dietary restriction for generations. Nevertheless, the expanding applications of IF research, from oncology and neurology to environmental medicine, signal the maturing of the field beyond its origins in metabolic and cardiovascular disease prevention [33,34,35,36,37].

Despite the progress documented in recent years, several important gaps remain. Mechanistically, the relative contributions of the fasting interval per se versus energy deficit, time-of-day effects, and macro-nutrient refeeding composition to the observed benefits are still inadequately separated in human studies. The chronobiological alignment of fasting windows with endogenous circadian rhythms represents a particularly fertile area, given emerging evidence that eating in the morning-to-early-afternoon window may be metabolically superior to an equivalent nighttime window [38,39].

Clinically, adequately powered long-term randomized controlled trials are urgently needed across multiple disease contexts, particularly oncology, neurodegenerative disease, and non-alcoholic fatty liver disease. Head-to-head comparisons of distinct IF protocols in the same patient population would substantially advance protocol selection guidance. The needs of vulnerable populations (older adults at risk of sarcopenia, individuals with a history of eating disorders, and patients on polypharmacy) warrant dedicated safety-focused investigation.

From a precision nutrition perspective, the development of reliable biomarkers, including gut microbiome signatures, plasma metabolomic profiles, and continuous glucose monitoring patterns, to predict individual response to IF protocols represents a high-priority translational objective. The integration of digital health technologies and wearable sensors into dietary intervention trials will likely accelerate progress in this direction [40,41,42,43,44,45,46,47,48].

Finally, the mechanistic synergism between IF and dietary quality should be rigorously tested in factorial trial designs that can quantify interaction effects. Understanding whether the benefits of IF are multiplicative, additive, or independent of concomitant dietary improvements is essential in the development of evidence-based combined nutritional strategies [49,50].

This Special Issue brings together a rich and diverse set of studies that collectively advance our understanding of intermittent fasting as a multidimensional biological intervention with broad therapeutic relevance. From appetite regulation and gut microbiome modulation to neuroprotection and cancer care, the contributions published herein reflect the expanding scope and increasing methodological sophistication of IF research.
